# Teaching Approaches to Learn Theoretical Contents in Physical Education: A Study about Contour Lines

**DOI:** 10.3390/ijerph17228599

**Published:** 2020-11-19

**Authors:** Nicolás Julio Bores-Calle, Ana Escudero, Daniel Bores-García

**Affiliations:** 1 Department of Didactics of Musical, Artistic and Corporal Expression, Faculty of Education, University of Valladolid, 34004 Palencia, Spain; nicolasjulio.bores@uva.es; 2 Department of Research and Psychology in Education, Faculty of Education, Universidad Complutense de Madrid, 28040 Madrid, Spain; anescude@ucm.es; 3 Department of Physical Therapy, Occupational Therapy, Rehabilitation and Physical Medicine, Health Faculty, Rey Juan Carlos University, 28922 Alcorcón, Spain

**Keywords:** teaching interventions, secondary education, theoretical contents, contour lines, orienteering

## Abstract

Purpose: Fostering student’s map reading skills, specifically understanding contour lines, is a challenging area of the Physical Education curriculum. Method: 238 students in their first year of secondary education (Mage = 13.1) were randomly assigned to one of these experimental conditions in physical education classes: (a) Teaching intervention 1 (TI-1): integrating the concept of contour lines into practical sessions of acrobatic gymnastics; (b) TI-2: theoretical sessions regarding contour lines; (c) Active control (AC): reading an introductory text about topographic maps; and two passive controls (PC) without any intervention, (d) PC-1 and (e) PC-2. Results: All students, except for PC-2, improved their knowledge of contour lines. Nevertheless, performing corporal figures (in TI-1) and employing pointing and tracing gestures (in TI-2) helped students to correctly resolve a broader range of tasks. Conclusions: The results highlighted the benefits of teaching proposals that favor movement and the experience of the body.

## 1. Introduction

With increased progress in technology, the ability to read and interpret topographic maps has become essential [1]. The possibilities to create and use these maps have been intensified as traditional practicalities of searching for a route or a place have been added to others that are included in what is called neogeography [2]. Consequently, there is an increased need to teach topographical skills and knowledge, an area that is usually reserved for specialized professions. Some authors and researchers point to schools as being responsible for providing the knowledge and the skills to read and interpret maps [3]. In fact, this content is mandatory in the Spanish curriculum of the compulsory secondary education within the areas of Geography, Biology, and Physical Education (PE) [4]. The approach to map usage as an educational content in all these areas entails the ability to understand and cope with the space in which we live, related to the ideal of keeping healthy and respecting the environment. In fact, both ideas comply with some of the principles of physical literacy [5,6]. 

Decoding the information hidden in a topographic map is not an easy task. It requires not only recognizing each of the symbols and identifying the different patterns that they can form, but also making more or less complex estimates and inferences about the terrain [7]. If the ultimate goal of topographic maps is spatial location and the search for routes or navigation through the terrain, something that can only be attained when a person has the skills and necessary understanding to use these maps, then it is essential to design educational proposals. These proposals should focus on both the conceptual and symbolic components of topographic maps [8]. This has sparked the search for diverse methodological solutions [9,10,11].

According to the literature, one of the main difficulties when interpreting topographic maps is that spatial elements are represented with two-dimensional, non-spatial symbols, such as contour lines [1]. Specifically, the correct interpretation of contour lines requires the combination of various skills related to spatial, logical, and symbolic reasoning, among others: the three-dimensional visualization of the shape represented by the lines, the understanding that a continuous dimension (such as height) is represented on the map categorically (with different lines), and the ability to relate the distance between those lines to the uneven surface (slope) of the plotted land [12]. 

The contour line concept is not only complex for high school and university students from different areas [1,13,14], but it is even challenging for people with map reading skills [10,15]. Due to the inherent difficulty in the interpretation of the contour lines, some works have been dedicated to presenting different strategies to try to teach it. For example, Segara et al. [3] have applied the principles of the socio-constructivist approach as the basis of a cartographic literacy model in schools. 

Others, such as Atit et al. [13], have addressed the design of instructional strategies for teaching contour lines from a more cognitive perspective, employing educational tools gestures, which symbolize or highlight spatial information, and the use of specific vocabulary. They evaluated the understanding of the shape and elevation of the terrain in female university students who were new to the subject using two experimental studies. In the first one, they observed that the pointing and tracing gestures of contour lines was the strategy that produced the best results, even greater than the three-dimensional representation of different shapes (hill, valley, or slope) with hands. The authors assumed that the pointing and tracing gestures helped learners to focus on the most relevant information and facilitated the abstraction of these visual patterns. In the second experiment, they showed that the type of verbal information given to the participants also influenced what was learned. For example, the group that had listened to specific vocabulary related to shapes was more successful in the exercises that evaluated this concept than in the rest of the exercises. From these studies, the instructional practice with the best results, at least with university students without previous knowledge, appears to be the one that integrates the two modalities: the one with pointing and tracing gestures of contour lines and the verbal one. With the current research, we intend to discover whether or not these results can be extrapolated to secondary school students.

At this point, from an educational point of view, it seems relevant to emphasize that the model described by Atit et al. [13] is not strange in a school context. First, the benefits of the use of gestures in the teaching-learning processes have been widely documented in Cognitive and Educational Psychology [16,17,18,19]. Second, its principles are in line with the principles of movement pedagogy, which is characterized by the recognition and use of the body and movement as fundamental axes of learning [20]. Finally, there are also great similarities with embodied cognition models. According to the latter, body, gestures, and actions play an essential role in human cognitive functions [21]. These theoretical assumptions underlie the hypothesis that the instructional practice described by Atit and her collaborators can also be effective with secondary school students.

A peculiarity in the teaching of PE is the traditional division between theoretical and practical lessons, which are normally taught in different spaces: a classroom is used for the teaching of theoretical content, whereas practical classes take place at the gymnasium [22]. This simplistic vision of PE has been overcome by those methodologies that integrate theoretical and practical content [23,24]. This is the reason why the study also aims to assess whether the idiosyncrasies in PE classroom can allow one to effectively teach important and complex concepts, such as that of contour lines, by integrating it into the practice.

The current research explores the integration of theoretical contents into practice within the framework of the Pedagogical Treatment of the Body teaching approach (of which its acronym is TPC, according to the Spanish name Tratamiento Pedagógico de lo Corporal) [25,26]. TPC is based on the idea that the body is not accidental but essential for being human, acting, and learning in human environments such as schools. It takes into account individual differences and tries to offer valuable corporal experiences to every student according to the criteria of relevance, equal opportunities, and polyvalence [27]. Moreover, TPC tries to avoid teaching a kind of PE of which its content is seen as consuming goods that are quickly outdated, with no other purpose than entertainment [28]. In this sense, TPC highly agrees with many of the principles of physical literacy [5,6].

This vision of PE intends that students: get a good relationship with their own bodies which involves respect, acceptance, and feeling confident; have good relationships with other people’s bodies, by accepting them and not feeling uncomfortable in their interactions; develop a set of basic motor patterns that allow students to perform various physical activities, and achieve a level of autonomy and knowledge to enable students to organize and manage healthy physical activities for further details, see [25].

Quite briefly, some of its main educational principles are: the active role of the person who learns, who is the reference center; the recognition of individual differences; the integration of cognitive, emotional, relational, and motor competences; the promotion of a taste for learning and a feeling of competence; and learning by realizing, not learning without realizing it [24]. So, lesson plans for secondary school are organized in increasing sequences of basic cycles of proposal-action-reflection-rethinking that base their development on pedagogical-didactic principles (taking precedence over classical anatomical-physiological principles). They are characterized by the search for a balance between the different types of content, diversifying and adapting the type of practical activities for this purpose, and using a student’s blank notebook. This notebook acts as a support for the teaching, learning, and evaluation process, which reaffirms the possibility that concepts can be learned through practical experiences [29].

Therefore, the objective of this study was to analyze the effect that different types of teaching interventions (i.e., integrating theoretical contents into practice or focusing on theoretical contents) have on learning to read topographic maps, specifically as it pertains to the understanding of the contour line concept (which includes notions such as the land’s shape, height, or slope), in students in the first year of Compulsory Secondary Education. This was educational research using a quasi-experimental design, which was conducted during real lessons of PE, contextualized within the secondary school curricula setting. The final objective was to present scientific evidence to facilitate teachers’ selection of appropriate methods to address certain curricular content [30]. In line with previous research, we expected that the groups of students who received the teaching interventions, both those that integrated the concepts into practice and those that were exclusively devoted to theoretical content, would obtain better results in the post-test than the control group participants.

## 2. Materials and Methods 

### 2.1. Design 

The first factor, between-subject, was the experimental condition. In other words, the type of teaching intervention carried out by the physical education teacher: Teaching Intervention 1 (TI-1) based on TPC (theoretical contents integrated into practice) where the contour line concept was worked on embedded in the acrobatic gymnastics teaching unit in PE classes (the acrobatic gymnastics teaching unit was chosen because it was the corresponding one, according to the educational planning, at the time the data were collected); Teaching Intervention 2 (TI-2) focused on the theoretical contents and the concept of the contour line was worked on in PE following a procedure similar to that described by Atit et al. [13]; Active Control (AC) condition, where the teacher read a brief summary to the class with basic information on topographic maps, without carrying out any additional teaching intervention (similar to the design by Atit et al., [13]); and two Passive Control (PC) conditions, where students did not receive any information about topographic maps in PE classes (PC-1 and PC-2). The second factor, within-subject, was the moment the test was applied: pre-test (assessment before working on the content) vs. post-test (after finishing the last intervention session).

It was a quasi-experimental study because the allocation of each of the participants to the different experimental conditions could not be totally random. Further, 10 classes of the first course of secondary education participated. Each class was randomly assigned to one of the experimental conditions.

### 2.2. Participants

The sample of the study was 238 students (112 girls; mean age = 13.1 years, *SD* = 3.3 months) from 10 different classes of the first year of a public secondary school in the northeast area of the city of Madrid. The division of classes established by the school center was retained. Thus, each intact class was randomly assigned to one of the experimental conditions: Teaching Intervention 1 (TI-1), which integrated the theoretical contents into practice; Teaching Intervention 2 (TI-2), which focused on theoretical content; Active Control (AC); and Passive Control (PC-1) (see Table 1). In order to have a measure that allowed us to control or rule out the influence of possible confounding variables that could affect the first data collection, a second Passive Control group (PC-2) was formed after approximately one year with two other groups of randomly selected first-year secondary students.

Permission was obtained from both the management of the school center and the families of all participating students before the data collection started. According to the information provided by the center, none of the evaluated students had learning difficulties, Spanish was the first language of all of them, their socio-economic status was middle, 98.7% were Caucasian (Spain’s majority ethnic group), and 1.3% were of gypsy ethnicity. None had previous experience in the field of orienteering and neither in reading topographic maps.

### 2.3. Materials

*Introductory text to topographic maps.* A short text (2 pages) was prepared with basic information in regard to the interpretation of topographic maps: a description of their usefulness and a brief explanation of the contour line concept. The material produced by Jacovina et al. [31], previously used in other investigations [13], was translated. The Spanish version was prepared by the authors and reviewed by a professional cartographer and agricultural engineer (JGC).

*Test of contour lines knowledge.* From the Topographic Map Assessment of Jacovina et al. [31], the authors, in collaboration with a professional cartographer, developed two tests to assess knowledge regarding contour lines, both adapted to the educational level of the participants in this study. All the questions were reviewed by a group of five people who were accustomed to the use of topographic maps (experienced mountain runners from the Spanish Orienteering Federation) or who create them (cartographers). These tests are available at: https://orcapalencia.com/web/pruebate/. This website belongs to the Spanish orienteering federated club O.R.C.A (Club de Orientación Río Carrión).

**Figure 1:**
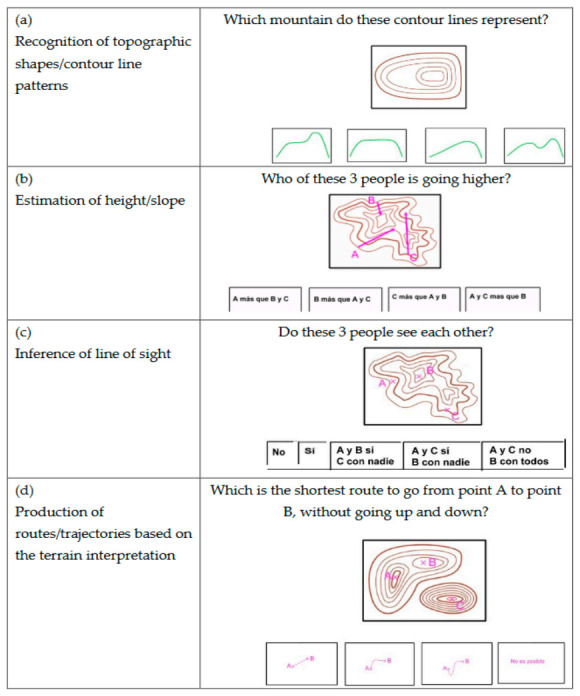
Selection of items from the final evaluation test (post-test).

The first test (pre-test), which aimed to assess the initial level of the participants, consisted of 11 questions. The second (post-test), which was applied at the end of the interventions, contained 30. The format of all the items was similar: a schematic image of a topographic element or a representation of contour lines with a question about it. All questions were multiple choice, with only one correct answer. Except for two items in the pre-test and two others in the post-test, four possible alternatives were presented plus the option to answer “I don’t know”. The questions involved at least one of the knowledge levels regarding contour lines described in previous studies [7,10]: (a) identify the topographic shapes represented by the lines; (b) estimate the height and/or slope of different elements; (c) infer the field of vision from some element; and (d) interpret the representations to define terrain paths or trajectories (see Figure 1).

The two tests, completed individually and with no time limit, were presented in paper and pencil format. The average duration of the pre-test was about 15 min and about 25 min for the post-test. In order to control the effects of the order in which the items were presented, three different orders were administered for the pre-test and four for the post-test and all of them were created and distributed randomly. 

Each correct answer received a score of one point. To achieve a general measure of the contour line knowledge, the scores of all the items that made up the test were added (see Atit et al. [13]). Thus, the maximum possible score was 11 and 30 points in the pre-test and post-test, respectively. 

The reliability analysis of the tests that evaluated the knowledge on contour lines showed that the two tests were reliable and showed good adequacy: α = 0.74 for the pre-test and α = 0.90 for the post-test. Another advantage of the tasks, which demonstrates their usefulness, was that they allowed a wide range of possible scores. Specifically, the diversity of scores in the pre-test was: Min = 0, Max = 11 (score in proportions = 1), *M* = 2.2 correct answers (*M*score in proportions = 0.20), *SD* = 2.2 (*SD* in proportions = 0.20). In the post-test: Min = 0, Max = 27 correct answers (score in proportions = 0.90), *M* = 14.6 answers (*M*score in proportions = 0.49), *SD* = 5.81 correct responses (*SD* in proportions = 0.20).

### 2.4. Procedure

**Figure 2:**
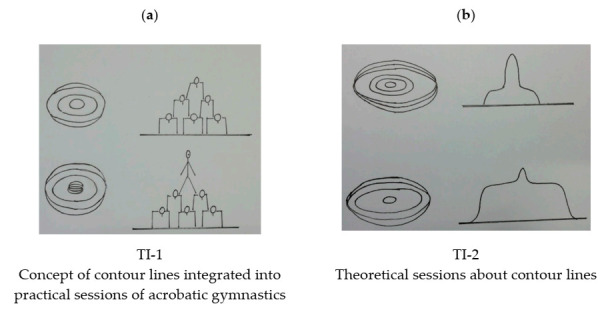
Selection of images shown to participants in TI-1 (**a**) and TI-2 (**b**).

The gathering of information began with the completion of the pre-test. The test was administered during the PE class. The teacher/researcher distributed the tests to all the students so that they could respond individually and remained present in the classroom to prevent the participants from exchanging comments. The teaching interventions began the following week (around five and seven days after). All of them were carried out by the same teacher (who was one of the researchers) during one or two sessions, depending on the type of intervention. They took place in the PE classroom, which had a projector and a whiteboard where the material from each intervention was projected from the computer. In the class immediately after the end of the intervention, the students completed the post-test under the same conditions as the initial evaluation (pre-test). The particularities of each of the educational interventions are described below:

*Condition 1. Teaching Intervention 1 (TI-1), theoretical contents integrated into practice* (based on the TPC, [25]). The total duration of the intervention was 50 min, distributed over 2 lessons. In the initial five minutes of the first lesson, the teacher/researcher read the introductory text to topographic maps, which was projected on the board. Next, he explained how the acrobatic gymnastics knowledge (that they were acquiring in the teaching unit that was underway) would help them understand the concept of contour lines (which involves notions such as shape, elevation, slope, and equidistance) through graphic representations such as those in Figure 2a. After the explanations (see Appendix A) and the corresponding feedback, the students worked in groups of 5–6 to represent different acrobatic gymnastics figures selected by the teacher/researcher. In the second lesson, the students were divided into work groups (5–6 members). The initial task consisted of physically representing the acrobatic gymnastics figure proposed by the teacher and then to use contour lines to represent it in each student’s notebook. The second task was the other way around: the teacher/researcher gave them a topographic representation to be represented as an acrobatic gymnastics figure both graphically and physically. The last one consisted of the students choosing a new figure, of their own invention, and representing it in the three modalities: graphically (in the notebook), physically, and topographically (contour lines). In order to avoid distorting the actual time spent on the intervention, the teacher/researcher controlled the time by means of a stopwatch to compare the duration of the intervention in the two classes that received it.

*Condition 2. Teaching Intervention 2 (TI-2), focused on the theoretical contents* (based on the procedure of Atit et al. [13]). The total duration of the intervention was 50 min. The first five minutes were devoted to reading the introductory text to topographic maps projected on the board. Next, the teacher/researcher showed five examples, such as those in Figure 2b, pointed out the important elements, and traced different shapes of the terrain. After discussing the concepts of shape, elevation, slope, and equidistance, the participants were asked to represent drawings of mountains using contour lines (see Appendix A). The teacher gave group feedback after this last exercise.

*Condition 3. Active Control (AC)* (based on the procedure of Atit et al. [13]). This lasted approximately 5 min. The teacher/researcher read the introductory text to topographic maps projected on the board. He provided no further information.

*Condition 4 and 5. Passive Control 1 (PC-1) and Passive Control 2 (PC-2).* Neither of these two groups received any information about topographic maps or contour lines in the PE sessions. Participants in this condition only completed the pre- and post-tests.

### 2.5. Data Analysis

The data analysis was performed with the statistical package SPSS v. 25. Before proceeding, the total scores obtained by the participants in the pre- and post-test were transformed into proportions, with the aim of making the levels of execution in both tests comparable. Due to the size of the sample (*n* > 40), it was possible to adopt the central limit theorem and perform parametric tests [32].

First, a series of preliminary analyses were performed, with Student’s *t* tests and one-way ANOVAs, to check whether variables such as the participant’s gender and the order in which items were presented affected the execution of the pre- and post-test. Likewise, a one-factor ANOVA was performed to demonstrate that the participants’ initial level of knowledge was equivalent across the different experimental conditions. Second, as the objective of the study was to examine the effect that different teaching interventions had on the understanding of contour lines, we carried out a mixed ANOVA 5 (Type of Intervention: TI-1 vs. TI-2 vs. AC vs. PC-1 vs. PC-2) × 2 (Evaluation moment: Pre-test vs. Post-test), with repeated measures in the last factor. There was a small number of missing data. A total of 15.9% of the participants only completed one of the assessment tests: 5.9% missed the pre-test session and 10% missed the post-test session; therefore, they have not been taken into account in the mixed ANOVA, although they have been considered in the rest of the analyses. 

## 3. Results

The preliminary analyses indicated that there were no significant differences based on gender or presentation order in the performance of the participants in the pre- and post-test. Likewise, the level of knowledge prior to the teaching interventions was equivalent in all participants, regardless of the type of intervention to which they had been assigned (see Table 2).

**Figure 3:**
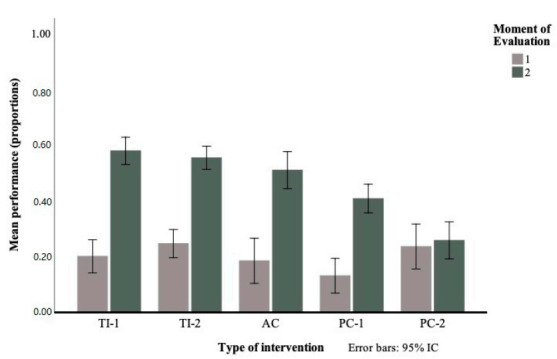
Interaction Type of Intervention x Moment of Evaluation.

The results of the mixed ANOVA indicated that the main effects of the Type of Intervention *F*(4197) = 7.188, *p* < 0.001, n_p_^2^ = 0.127 and Moment of Evaluation *F*(1197) = 356.589, *p* < 0.001 n_p_^2^ = 0.644, as well as the interaction Type of Intervention x Moment of Evaluation *F*(4197) = 16.121, *p* < 0.001, n_p_^2^ = 0.247 (see Figure 3) were all significant. 

In order to conduct a more in-depth analysis of the interaction, two unifactorial ANOVAs were performed, one for each level of the Moment of Evaluation factor. The results of these analyses of variance were not significant when considering the scores of the different groups in the pre-test, as indicated by the preliminary analyses, *F*(4218) = 1.647, *p* = 0.163, n_p_^2^= 0.029, but in the post-test, they were significant *F*(4216) = 19.316, *p* < 0.001, n_p_^2^ = 0.267. Bonferroni post-hoc tests showed the existence of significant differences between the scores achieved in the post-test by the participants of the TI-1, theory integrated into practice, and the Passive Control groups 1 and 2 (*p* < 0.001 in both cases). Similarly, significant differences were also observed between the performance of the students who received TI-2, focused on theoretical content, and those assigned to the two passive control groups (*p* = 0.001 and *p* < 0.001, Passive Control 1 and 2, respectively). In accordance with the hypothesis proposed, the data indicate that the final level of knowledge demonstrated by the students who received some of the interventions was significantly higher than that achieved by the students who received none. However, according to the analysis, the performance in the post-test of the Active Control group, who only listened to the reading of the introductory text regarding topographic maps, was similar to that obtained by the TI-1 and TI-2 groups and statistically higher than that achieved by Passive Control 2 (*p* < 0.001), which refines the starting hypothesis.

To explore the variations between the participants’ performance in the pre-test and in the post-test in each of the teaching interventions, paired samples *t* tests (Student’s) were carried out. The analyses indicated that the post-test scores were significantly higher than the pre-test scores in all cases, except in Passive Control 2, as is shown in Table 3. Contrary to expectations, even the Passive Control 1 group, which did not receive any information regarding contour lines, significantly increased their performance in the post-test. In order to delve into the underlying reasons for this improvement in the groups that had not received any teaching intervention (i.e., AC and PC-1), the following additional analyses were carried out.

**Figure 4:**
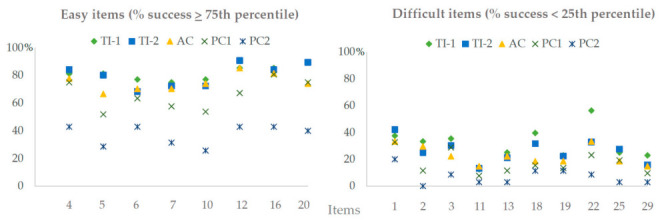
Percentage of participants, as a function of intervention type, who correctly responded to either easy or difficult items in the post-test.

Firstly, the possibility that the mere performance of the pre-test could serve as practice or learning was examined. The analyses indicated that this is not the case, given that the students who had not completed the pre-test (*n* = 15, *M*score = 0.49, *SD* = 0.19) obtained the same level of success in the post-test as those who did the pre-test (*n* = 202 in the whole sample, *M*score = 0.49, *SD* = 0.24): *U* = 1509, z = −0.026, *p* = 0.980. Specifically, all participants in the AC group responded to the pre-test. In PC-1, participants who did not carry out the pre-test: *n* = 6, *M*score = 0.50, *SD* = 0.17; who did pre-test: *n* = 42, *M*score = 0.40, *SD* = 0.16. *U* = 86.5, z = −1.24, *p* = 0.217. Second, the diversity of the scores obtained in the post-test was evaluated. The participants’ levels of success varied depending on the demands from the different items. An exploratory analysis has shown that the easiest items, those that were answered by at least 75% of the participants, consisted of identifying the topographic figures represented in the contour lines. However, in four of them (items 5, 7, 8, and 9), the number of students in the passive control groups (PC-1 and PC-2) who answered them correctly was lower than that of the other groups: χ^2^ (4) = 37.77, *p* < 0.001; χ^2^ (4) = 22.41, *p* < 0.001; χ^2^ (4) = 11.05, *p* = 0.026; χ^2^ (4) = 30.41, *p* < 0.001, in items 5, 7, 8, and 9, respectively (see Figure 4). In regard to the more complicated questions (with success rates below the 25th percentile), which involved inferring routes or trajectories and estimating points of view, it was the students who received one of the two teaching interventions who performed best, as can be seen in Figure 4. The case of questions 18 and 22 stands out, in which the greatest number of correct answers were found in the intervention that integrated theory into practice (TI-1): χ^2^ (4) = 13.72, *p* = 0.008; χ^2^ (4) = 23.78, *p* < 0.001, in items 18 and 22, respectively.

## 4. Discussion and Conclusions

This study analyzed the effect that different teaching interventions have on the understanding of contour lines, one of the most complex elements in the reading and interpretation of topographic maps [10,15]. Two teaching interventions in PE were proposed to students from their first year of secondary school. One was based on the TPC model (TI-1), which integrated the theoretical contents into practical sessions. The other dealt exclusively with theoretical content (TI-2) and adapted the proposal of Atit et al. [13], whose validity had been demonstrated in their empirical study, to the school context. Several control groups were also included: the AC, which allowed the comparison of the current results with the literature (e.g., [12,13]), and two passive control groups.

As expected, the data reveal that the two teaching interventions, both the one that integrates the theoretical contents with practical experience and the one focused on theoretical concepts, increased the students’ knowledge of contour lines. Similarly, we found an equivalent increase in students in the AC group, caused by simply reading the introductory text. These findings support those obtained by Atit et al. [13] and are consistent with the proposals from the field of psychology. On the one hand, they underline the importance of language in learning processes and, in particular, of spatial terms for the development of spatial knowledge. There is wide evidence that supports that the acquisition of this specific vocabulary helps people to construct conceptual representations, which allow people to understand spatial concepts [33,34]. 

On the other hand, these data also coincide with the proposals that suggest that seeing gestures, and repeating them, support these abstraction processes [17,35]. They also align well with the results from Jaeger et al. [36], which showed that the instructional approaches that provide grounding activities in embodied experience facilitate learning in science; and with those from Cecchini and Carriedo [37], who suggested that the integration of physical activity into other subjects, such as mathematics, could contribute to increased learning in both areas.

Furthermore, all the students evaluated during the first gathering of information, including those from the PC-1, showed an increase in their knowledge level compared to the baseline. The improvement in the experimental and active control groups is attributed to what was mentioned in the previous paragraph, but what could have caused it in the passive control group? What type of influences, other than the interventions, could affect the results of the post-test? We are considering various hypotheses.

The first could be related to a practice effect: a positive effect from taking pre-test in participants’ performance at responding to the post-test. There is literature that focuses on the benefits that testing or assessments offer in the acquisition and retention of knowledge [38,39,40]. However, our data do not support these possibilities given that the level of success achieved in the post-test was similar in students who did answer the pre-test compared to those who did not. Furthermore, in the PC-2, the level of knowledge shown by the students in the initial test was similar to that observed in the final test.

The second hypothesis considers that this improvement, achieved in the absence of a specific intervention, may have to do with variables inherent in the normal operation of an educational center, beyond the control of the experimenters. Confident that content related to topography was not formally taught during this period in any other subject, we attribute this increase in performance to the fact that learning processes do not occur exclusively within classrooms, nor are they always supported by the teacher-student relationship [41]. We understand that the environment of interest created in the center provoked new and richer social interactions between students, including those in the PC-1 group, which led to informal learning that was less structured, without teacher control, and resulted from interactions in which the students did not consciously expect to learn [42,43,44]. We highlight the social dimension in this type of learning, which would include seeking help, exchanging information, or peer feedback. In a recent study, novice teachers considered that the feedback that was given between colleagues, informally, was especially relevant for the development of their professional competences [45].

With the intention of controlling these possible extraneous variables, we decided to gather information again the following year with a new control group (PC-2) in the same educational center, following an identical procedure and with the same teacher/researcher. On this occasion, the level of knowledge shown by the students, in the absence of intervention, was similar in the initial and final evaluation. This result supports the effectiveness of teaching interventions and the idea of informal peer learning outside the classroom.

However, doubts may arise about the utility of the teachers’ effort to plan and develop systematic teaching-learning processes regarding the interpretation of contour lines. In order to dispel any doubts, some specific details must be clarified. Although there were general improvements in four out of the five groups, not all learning was the same since not all questions involved the same difficulty. Compatible with the taxonomy proposed by Rautenbach et al. [10], the questions that involved identifying the figures represented from contour lines were also the easiest for the participants of the current study. Similarly, those that required a higher degree of abstraction, such as the estimation of heights or the ability to navigate when defining routes or trajectories, were the most complex (see also Ooms et al. [1]).

Despite the improvements shown by the mean scores, the performance of the participants in the PC-1 group was always lower than that achieved by those who had received instruction, both in the simple and the difficult questions. On the other hand, the students whose intervention integrated theoretical concepts into practice (based on the TPC) were the ones who most frequently answered the difficult questions. According to these findings, it seems that there is a more basic type of knowledge, which is easy to assimilate, can be acquired with simple tasks, and requires little dedication from the teacher, such as simply reading a text adapted to the participants. However, such material would not be sufficient to cognitively solve more complex tasks, which require a certain level of reasoning. For this, more complex teaching strategies are required in which the active and conscious intervention of the student and the teacher are necessary for their assimilation and understanding. Undoubtedly, knowing what type of learning (or levels of knowledge) each teaching proposal favors is essential in order to propose effective educational strategies. 

According to the premises of the TPC, cognitive processes take place with the whole body, where movement and the experience of the body itself promote conceptual understanding [25]. We believe that this is what has happened in the TPC teaching proposal analyzed here: embedding the theoretical content in the corporal dimension by means of acrobatic gymnastics figures has facilitated student’s learning. We find the results of the TI-2 to be similar, since it also uses the body and movement [20,21]. The difference, which is an advantage, is that the TI-1 eases the problems of the theory-practice division that as Hernández-Álvarez et al. [46] indicates, involves loss of time and a lack of interest in the theoretical contents by the teaching staff in PE. In addition, learning can occur simultaneously with other contents different from those of orienteering and with different objectives and evaluation criteria. In the case of this TPC proposal, the students moved between the acrobatic gymnastics and orienteering units along what seems to be an impossible path, but which takes on meaning as the connection is made between the body in motion and the two-dimensional representation of the terrain.

Even so, the results of this study should be taken with caution. It was educational research using a quasi-experimental design in the school curricula setting, which can explain some of its limitations in comparison with lab experiments, such as sample characteristics or teachers’ involvement in the research. Undoubtedly, the particular characteristics of educational contexts considerably hinder the possibility of controlling all the confounding variables, especially those that have to do with the exchange of information among students. Despite these limitations, from our point of view, carrying out interventions in a systematic way, in real classroom situations, positively affects the ecological validity of this study.

In conclusion, the results of this study highlight the effects of teaching proposals that favor the conceptual understanding of contour lines. This may offer benefits for researchers and educators to improve learning outcomes and design interesting educational strategies for the school context. Lastly, the present investigation opens up the possibility of future paths of explorations. It would be interesting to see if the results can be replicated with students from different educational contexts and of different ages. Likewise, the long-term permanence of this learning and its transfer to the actual reading of maps in everyday life or in sports should be explored. Perhaps they are key criteria for selecting the most appropriate teaching methodology. In addition, interdisciplinary approaches to teach map reading skills as cross-curricular content appear as promising ways of doing that and should be further studied. They may foster students to develop healthier lifestyles in which they interact with the environment with safety, respect, and responsibility.

## Figures and Tables

**Table 1:** Sample characteristics as a function of the intervention type.

Characteristics		TI-1	TI-2	AC	PC-1	PC-2
Number of classes randomly assigned		2	3	1	2	2
N total (students)		48	76	27	52	35
Gender	Girls Boys	24 24	32 44	15 12	25 27	16 19
Mean age (y.o.) (*SD* in months)		13.1 (3.7)	13.1 (2.7)	13.1 (2.3)	13.1 (2.3)	13.2 (4.6)

Note: TI-1: theorical content integrated into practice; TI-2: theorical session; AC: active control; PC-1: passive control; PC-2: second passive control, with data collected one year later than the rest.

**Table 2:** Means and standard deviations of preliminary analyses: Correct responses in the pre- and post-test as a function of gender, presentation order, and intervention type.

Variable	Moment of Evaluation							
	Pre-Test				Post-Test			
	*n*	*M*	*SD*	IQR	*n*	*M*	*SD*	IQR
Gender								
Boys	116	0.19	0.19	[0.00–0.73]	114	0.48	0.18	[0.00–0.87]
Girls	107	0.20	0.22	[0.00–1]	103	0.49	0.21	[0.03–0.90]
Presentation order								
1	90	0.22	0.21	[0.17–0.26]	63	0.47	0.20	[0.42–0.52]
2	82	0.18	0.20	[0.14–0.23]	67	0.46	0.21	[0.41–0.51]
3	51	0.19	0.19	[0.13–0.24]	45	0.54	0.19	[0.48–0.60]
4	-	-	-		42	0.50	0.16	[0.45–0.55]
Intervention type								
TI-1	48	0.20	0.20	[0.14–0.26]	-	-	-	-
TI-2	67	0.24	0.19	[0.20–0.29]	-	-	-	-
AC	27	0.18	0.23	[0.09–0.26]	-	-	-	-
PC-1	46	0.15	0.18	[0.10–0.20]	-	-	-	-
PC-2	35	0.19	0.24	[0.11–0.27]	-	-	-	-

Note: TI-1: theorical content integrated into practice; TI-2: theorical session; AC: active control; PC-1: passive control; PC-2: second passive control, with data collected one year later than the rest. *n*: sample size; *M*: mean score (of correct responses); *SD*: standard deviation; *IQR*: interquartile range 95% confidence interval.

**Table 3:** Results of paired samples *t* tests examining the difference between participants’ performance in pre- and post-test as a function of the type of intervention.

Intervention Type	*n*	*M*Score (*SD*) Pre-Test	*M*Score (*SD*) Post-Test	Student’s *t*	df	*p*	95% CI	Cohen’s *d*
TI-1	46	0.20 (0.20)	0.58 (0.17)	−14.65	45	<0.001	[−0.44, −0.33]	2.02
TI-2	64	0.25 (0.19)	0.55 (0.15)	−12.191	63	<0.001	[−0.36, −0.26]	1.784
AC	25	0.19 (0.23)	0.51 (0.17)	−10.488	24	<0.001	[−0.38, −0.25]	1.581
PC-1	42	0.13 (0.17)	0.41 (0.16)	−10.456	41	<0.001	[−0.33, −0.22]	1.659
PC-2	25	0.24 (0.26)	0.26 (0.21)	−0.552	24	0.586	[−0.11, −0.06]	0.095

Note: TI-1: theorical content integrated into practice; TI-2: theorical session; AC: active control; PC-1: passive control; PC-2: second passive control, with data collected one year later than the rest. *n*: sample size; *Mscore*: mean score (of correct responses); *SD*: standard deviation df: degrees of freedom; *p*: *p* value; 95% CI: 95% confidence interval.

## Appendix A

### Appendix A.1. TI-1 Concept of Contour Lines Integrated into Practical Sessions of Acrobatic Gymnastics

“The more curves there are, the higher the mountain will be. Look at these two figures (Figure 2a) [projected on the board]: Do you see that the pyramids [human figures of acrobatic gymnastics] are not equally tall? The first is higher because the person who is on the top is standing, instead of kneeling. Do you see that figure has more contour lines than the other? That is the reason”.

“The distance between one curve and another is always the same, and that is decided by whoever makes the map. In our case, each contour line will be a joint: the first will be the feet, the second the knees, the third the hips, the fourth the shoulders and the fifth the head with the arms extended upwards. So, if we want to represent a student standing with his arms up, we will have to draw five contour lines”.

### Appendix A.2. TI-2 Theoretical Session

“How many lines do you see in this image? [Teacher points and traces contour lines]. If the distance between lines is 20 m, for example, how high is the top of the mountain? [Teacher leaves time for the students to answer]. If there are 6 lines and in each line, we go up 20 m, we just have to multiply 6 × 20. The result is 120 m, which will be the height we would reach if we ascended the mountain to the top [trace the route]”. Teacher continues with the following example: “Would this mountain be higher or lower than the previous one? How many meters lower is it?”.
